# A Comparative Study of PLSR and SVM-R with Various Preprocessing Techniques for the Quantitative Determination of Soluble Solids Content of Hardy Kiwi Fruit by a Portable Vis/NIR Spectrometer

**DOI:** 10.3390/foods9081078

**Published:** 2020-08-07

**Authors:** Shagor Sarkar, Jayanta Kumar Basak, Byeong Eun Moon, Hyeon Tae Kim

**Affiliations:** Department of Bio-Systems Engineering, Gyeongsang National University (Institute of Smart Farm), Jinju 52828, Korea; shagor.sarkar@gnu.ac.kr (S.S.); basak.jkb@gmail.com (J.K.B.); be25moon@naver.com (B.E.M.)

**Keywords:** hardy kiwi, near-infrared spectroscopy, non-destructive measurement, partial least square, support vector machine, soluble solids content

## Abstract

Linear partial least square and non-linear support vector machine regression analysis with various preprocessing techniques and their combinations were used to determine the soluble solids content of hardy kiwi fruits by a handheld, portable near-infrared spectroscopy. Fruits of four species, namely Autumn sense (A), Chungsan (C), Daesung (D), and Green ball (Gb) were collected from five different areas of Gwangyang (G), Muju (M), Suwon (S), Wonju (Q), and Yeongwol (Y) in South Korea. The dataset for calibration and prediction was prepared based on each area, species, and in combination. Half of the dataset of each area, species, and combined dataset was used as calibrated data and the rest was used for model validation. The best prediction correlation coefficient ranges between 0.67 and 0.75, 0.61 and 0.77, and 0.68 for the area, species, combined dataset, respectively using partial least square regression (PLSR) method with different preprocessing techniques. On the other hand, the best correlation coefficient of predictions using the support vector machine regression (SVM-R) algorithm was 0.68 and 0.80, 0.62 and 0.79, and 0.74 for the area, species, and combined dataset, respectively. In most cases, the SVM-R algorithm produced better results with Autoscale preprocessing except G area and species Gb, whereas the PLS algorithm shows a significant difference in calibration and prediction models for different preprocessing techniques. Therefore, the SVM-R method was superior to the PLSR method in predicting soluble solids content of hardy kiwi fruits and non-linear models may be a better alternative to monitor soluble solids content of fruits. The finding of this research can be used as a reference for the prediction of hardy kiwi fruits soluble solids content as well as harvesting time with better prediction models.

## 1. Introduction

Fruits are manually or mechanically sorted based on size, color, shape, and surface defects, while internal quality parameters including soluble solids content (SSC), acidity (pH), vitamins, and phytochemicals are generally determined by destructive approach [1]. Regarding kiwi fruit production, the determination of internal quality attributes is a paramount concern to consume quality fruits. In recent years, the improvement of non-destructive technology has gained much attention for the application in measuring food quality, which is easy in operation, fast, and reliable than traditional methods [2]. Visible/near-infrared (Vis/NIR) spectroscopy is one of the promising nondestructive analytical methods, which does not require any pre-sample preparation for assessing the quality. Several studies focused on the application of Vis/NIR as nondestructive quality measurement of apple, mango, citrus, and kiwi fruit. For apple, reflectance spectra of 800–1600 nm were obtained to predict soluble solids content by normalizing the spectral reflectance with a standard error of predictions (SEP) [3]. To evaluate the organoleptic properties, the absorbance spectra of the near-infrared spectroscopy (NIRS) was used to correlate the consumer preference or sensory panels [4] and internal qualities [5,6,7,8,9]. NIR also used to predict the optimal harvesting date of apple [10] and to distinguish the fruit in storage condition [11]. In the case of mango, the correlation between the NIR range and various internal quality parameters was also established [12]. For sorting ripe kiwi fruit, a suitable preprocessing method has been developed to predict a sugar content model of hardy kiwi by NIR spectroscopy based on various internal parameters and for the verification of a developed low-cost sensing system [13,14].

In the ripening stage of kiwi fruit, insoluble starch is converted into soluble solids increasing sugar content, whereas flesh firmness and acidity decrease in parallel [13]. The internal quality conversion occurs before the fruit reaches eating ripeness is known as physiological maturity of fruit at harvest [15]. However, SSC is a major quality-determining parameter and linked to consumer taste preference [16,17]. In the application of Vis/NIR spectroscopy for the prediction of internal quality and developed models for predicting SSC of kiwi fruit, principal component analysis (PCA) was used for *Actinidia deliciosa* (A. Chev) [13], partial least square regression (PLSR) was used for Qinmei kiwi fruit [18]. In order to establish a relation and model between SSC and spectra, some non-linear variations, and artificial neural network (ANN) have been used in a previous study [19]. The PLSR results in the most reliable and robust model for SSC and other internal quality parameters as well. Moghimi et al. investigated the NIR spectroscopic (400–1000 nm) technique with PLS analysis with several preprocessing methods to determine SSC (RMSEP = 0.295) and acidity (RMSEP = 0.076) of hayward kiwi fruity [20]. McGlone et al. used VNIR spectra (300–1140 nm) with a 3.3 nm sampling interval for predicting dry matter (DM) and SSC of *Actinidia chinensis* Planch var. chinensis “Hort16A” with density, where PLSR method was applied to compare the results [21].

Experimental conditions and instrument variations, analyzing characteristics influence non-linearities in spectra. Regarding this kind of situation, non-linear models may contribute a more optimal solution than other classical multivariate calibration models [22]. SVM has already been introduced as an alternative to existing methods for regression and classification. Green, black, and oolong teas were identified using SVM as an application of chemometrics [23]. Du et al. [24] investigated the eating ripe stage and SSC of *Actinidia chinensis*. cv. Hongyang using SVM-R, while physical properties were estimated from NIR spectra of wood using SVM [25].

As NIRS is developed in response to the speed in analysis and flexibility in adapting to different fruit samples, it provides a large amount of spectral data that consists of noise and affected by several physical, chemical, and spectral variables. Also, it is challenging to categorize spectral information between samples [26]. As a solution to these problems, chemometrics was introduced for extracting information from noisy data which contains different preprocessing techniques, multivariate calibration methods, and variable reduction methods to produce accurate and reliable models [20]. Fan et al. [27] used the second derivative preprocessing technique to develop a model for the prediction of SSC and firmness with correlation coefficient R^2^ of 0.9532 and 0.8136 with a standard error of prediction (SEP) of 0.3838 and 0.5344, respectively for Red Fuji apples. Muik et al. [28] used PLSR for the validation of a model of pomace to determine oil and water content with different pre-treatments, including mean centering, variance scaling, multiplicative signal correction (MSC), Savitzkye-Golay smoothing, and first and second derivatives to compare the effect of each preprocessing techniques.

Chemometrics methods are needed to analyze the spectra characteristics using a set of calibration methods. The most common and generalized analysis for chemometrics is PLS, which is mostly employed in Vis/NIR spectroscopy analysis [29]. PLS regression is an effective tool to model the linear relationship between the multivariate predictor X (spectral data) and response variables Y (the properties of interest) [30,31] and the most significant properties of SVMs are their ability to take care of large input spaces efficiently, to manage noisy patterns and multi-modal class distributions, and their restriction on a subset of training data to suit a non-linear function [32].

Studies carried out on hardy kiwi fruit with NIRS include heavy, equipped, complex, and lab-based NIR systems. Hence, a portable, user-friendly, and handheld NIR spectrophotometer is highly capable of in situ measurements that will help kiwi growers to check the pre-harvest maturity level and internal fruit attributes in a non-destructive way [33]. Based on the above discussion, this research was designed with an aim to compare the results of two different analyzing procedures PLSR and SVM-R with various preprocessing techniques for the prediction of SSC of hardy kiwi fruit from NIR spectra acquired using a portable handheld spectrometer.

## 2. Materials and Methods

### 2.1. Sample Preparation

The hardy kiwi fruit (*Actinidia arguta*) was used in this study and collected from ten different farms of five regions in South Korea. Table 1 shows the location of each farm in five areas as follows:

In this study, hardy kiwi samples were collected from five areas (Gwangyang, Muju, Suwon, Wonju and Yeongwol) which include four species, namely, Autumn sense, Chungsan, Daesung, and Green ball. All samples were subjected to a post-ripening process under same environmental conditions of temperature (20 ℃), and relative humidity (65%) for three days. Datasets were prepared based on area, species, and in combination. Area dataset includes samples data of each area, species dataset includes different farms data, and combine dataset includes all data together. The classified dataset of each area, species, and combine set are presented in Table 2.

Afterward, datasets were divided into two groups equally for each area, species, and combine set: the first group was used to develop the calibration models and the other for predicting quality and model validation purposes.

A number of factors, such as geographical origin, temperature, season, cultivar, and fruit maturity affect the fruit cellular and optical structure as well as chemical composition and fruit internal quality parameters, such as SSC, moisture, and acidity [34]. Therefore, to establish a more reliable and robust model for the prediction of SSC, biological and physical changes in samples on the NIR spectrum should be considered. In this study, one approach was applied to develop models using individual area data. The individual area or origin model indicates more reliable prediction ability if the predicted samples were collected from the same area [35]. Furthermore, a prediction model depending on a variety or species is more appropriate than the multiple species [36]. On the contrary, multiple species or varieties-dependent dataset gives a more accurate and robust prediction model for the determination of harvesting time or fruit maturity [37]. By considering all these integrities, datasets and the models were developed for each area, each species, and in combination.

### 2.2. Spectra Acquisition

In this experiment, spectra acquisition was based on interactance mode; each sample was scanned using a portable, handheld, and low precision near-infrared spectrophotometer (F-750 produce quality meter, Felix Instruments, Inc., 1554 NE 3rd Avenue Camas, WA, USA). The Vis/NIR spectrophotometer scans four times simultaneously to produce one average absorbance spectra by log (1/R reflectance) of the intact fruit between the wavelengths of 310 and 1100 nm at a data resolution of 3 nm, spectral resolution ranging from 8 to 13 nm. Xenon tungsten light source was used in this instrument to illuminate the fruit through a sampling window of 30 mm diameter. The device equipped with a Zeiss MMS1 VIS-NIR spectrometer (Carl-Zeiss-Promenade 1,007,745 Jena, Germany) placed just behind the sampling window to incorporate the light transmitted through the skin and flesh of the fruit. Furthermore, absorbance spectra were converted to second derivative spectra using F750 Data viewer (Felix Instruments, Inc., 1554 NE 3rd Avenue Camas, WA, USA) software, and spectra from 729–975 nm were analyzed while 310 to 728 nm and beyond 975 nm were eliminated because of the noise containing in this wavelength regions.

### 2.3. Reference Measurement

Reference measurement of SSC of hardy kiwi is the primary internal quality attribute that was measured using a portable digital refractometer (SCM-1000, HM Digital Inc. 2629 Manhattan Beach Blvd. Redondo Beach, CA 90,278 USA) with a measurement range of 0–55%brix destructively after spectra acquisition with the spectrophotometer.

### 2.4. Chemometrics

The most important chemical ingredient of fruits and vegetables is water which absorbs near-infrared radiation [38], and near-infrared spectrum consist of a large set of overtones and combination bands which are convoluted because of the complicated chemical composition of typical fruits and vegetables [20]. In addition, spectrums can be convoluted by complicated wavelength-dependent scattering effects, tissue heterogeneities, instrumental noise, ambient effects, and other sources of variability [39]. Therefore, chemometrics would be the compatible solution to extract as much information from the interpretive NIRS data [26].

### 2.5. Spectral Data Preprocessing

**Figure 1:**
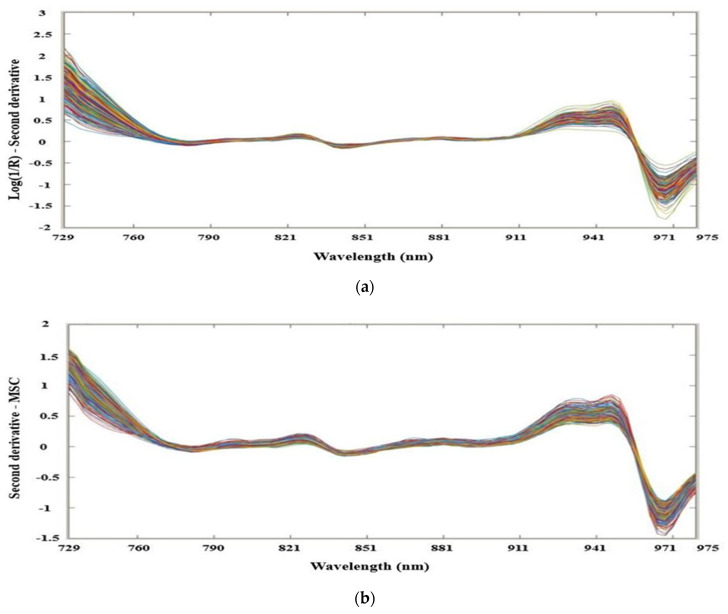

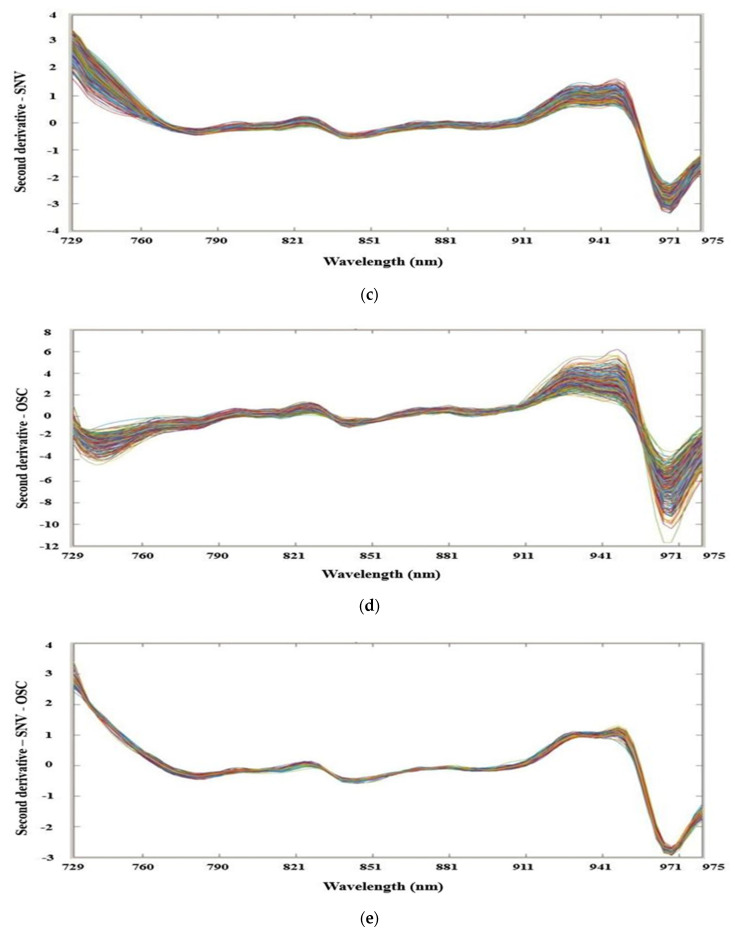

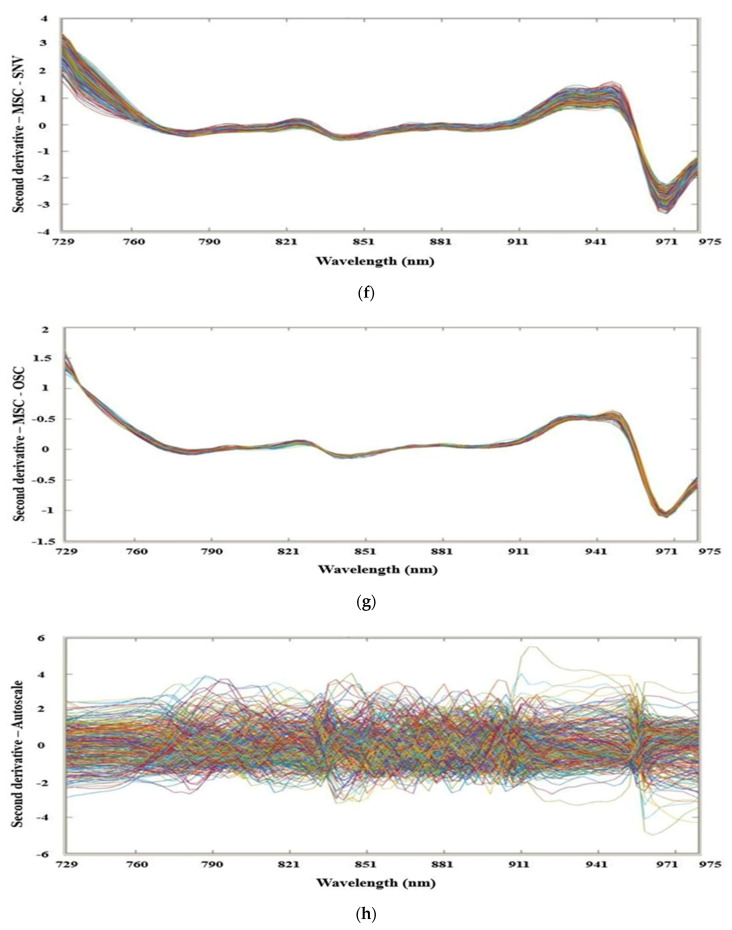
(**a**) Second derivative (SD) spectra; (**b**) spectra by SD + MSC; (**c**) spectra by SD + SNV; (**d**) spectra by SD + OSC; (**e**) spectra by the combination of SD, standard normal variate (SNV), and orthogonal signal correction (OSC); (**f**) spectra by the combination of SD, multiplicative signal correction (MSC), and SNV; (**g**) spectra by the combination of SD, MSC, and OSC; and (**h**) spectra by the combination of SD and Autoscale, from hardy kiwi samples.

The spectra data obtained from the spectrometer consist of background information and noise other than sample information, and it is very decisive to pre-process spectra data before modeling to obtain reliable, accurate, and steady calibration models [39]. Spectral preprocessing techniques are required to remove any irrelevant information, including noise, uncertainties, variability, interactions, and unrecognized features. In this study, original spectra were converted to second derivative preprocessing (SD), later several preprocessing methods such as normalization-multiplicative scatter correction (MSC), standard normal variate transformation (SNV), orthogonal signal correction (OSC), Autoscale, and combination of normalization preprocessing techniques were applied on second derivative spectra data to build calibration and prediction models. Second derivative preprocessing was used to remove background and increase spectral resolution and to remove slope additive baselines. Normalization removes the multiplicative spectral effects where the spectral vector transformed into unit length. Precisely, MSC preprocessing removes the ideal linear scattering and its effects, SNV removes the deviations caused by particle size and scattering, and OSC preprocessing removes the unnecessary signals before calibration and reduces the principal component (PC) number significantly which strengthens the prediction ability of the model [14]. Another preprocessing method Autoscale is used to remove the influence of variables measured on different scales [40] that make the data more suitable for data analysis. In addition, different preprocessing techniques were used for both the analysis of partial least square regression (PLSR) and support vector machine regression (SVM-R) for the comparison of predicted models. Various preprocessing techniques are used in this study, as shown in Figure 1.

### 2.6. Principle Component Analysis (PCA) and Partial Least Squares (PLS) Analysis

Before PLS analysis, PCA was used as a quantitative method to show the statistical variance among the different datasets. PCA loading values were obtained to select the impactful wavelengths within the selected window. The PCA was carried out considering only a part of the dataset.

Latent variable (LVs) is an essential factor that could be extracted from PLS according to the relevant predicted Y variable from a highly correlated and collinear original spectra. The number of LVs is very significant to build a PLS model, which is generally optimized by cross-validation of the calibration samples. Therefore, the optimal number of LVs was chosen using 10-fold contiguous block cross-validation until the root mean square of cross-validation (RMSECV) becomes a minimum. The PLS analysis was performed using MATLAB 2018b (MathWorks, Natick, MA, USA) with PLS Toolbox (Eigenvector Research Inc., 196 Hyacinth Road, Manson, WA 98831, USA).

The model performance was evaluated and calculated based on the following parameters: the correlation coefficient (R^2^) of calibration and prediction, root mean square errors of the calibration and prediction (RMSEC, and RMSEP) (see Equations (1) and (2), respectively), bias and ratio of prediction to deviation (RPD) (see Equation (3)) were used to test the predictability of the developed model. RPD calculation of the model suggests the accuracy of the prediction model, and the performance of the prediction model for NIR analysis can be classified as high, average, or low values of >2.0, 1.4–2.0, and <1.4, respectively where the higher RPD value indicates higher accuracy of the prediction [41,42].

(1) $$\mathrm{RMSEC}=\sqrt{\frac{\sum_{i=1}^{m_{c}}{{(y}_{i}-{\hat{y}}_{i})}^{2}}{m_{c}-k-1}}$$

(2) $$\mathrm{RMSEP}=\sqrt{\frac{\sum_{i=1}^{m_{p}}{{(y}_{i}-{\hat{y}}_{i})}^{2}}{m_{p}-1}}$$

where m represents the number of validation samples, the number of free variables is f, $y_{i}$ is the measured sugar content of sample, and ${\hat{y}}_{i}$ is the predicted sugar content of the sample.

(3) $$\mathrm{RPD}=\frac{\mathrm{SD}}{\mathrm{RMSEP}}$$

where SD is the standard deviation of the measured sugar content of validation set.

### 2.7. Support Vector Machine (SVM) Analysis

The SVM is a strong non-linear multivariate algorithm and establishes a hyperplane or set of hyperplanes in a high dimensional space for classification, and the same principle is used for the regression [43]. Three standard kernel functions, namely polynomial, radial basis, and sigmoid kernel function are used to build the SVM model, where radial basis function (RBF) is mostly used because of its capability of handling linear and non-linear relationships between the class labels and spectra data. Also, RBF can reduce computing intricacy of the training set, thereby providing an excellent performance under general smoothness assumptions [44,45,46].

In SVM-R, the cost value was used to optimize SVM model, and the optimum SVM-R model was achieved according to the minimum cost value. In this study, SVM-R was applied by choosing the ε-SVR (epsilon-support vector regression) algorithm with the following parameters: radial basis function (rbf) as kernel type, γ = 0.01 and ε = 0.1. MATLAB 2018b (MathWorks, Natick, MA, USA) with PLS Toolbox (Eigenvector Research Inc., 196 Hyacinth Road, Manson, WA 98831, USA) was used for the analysis of all datasets [47].

## 3. Results

### 3.1. Characteristics of Spectral Profiles

**Figure 2:**
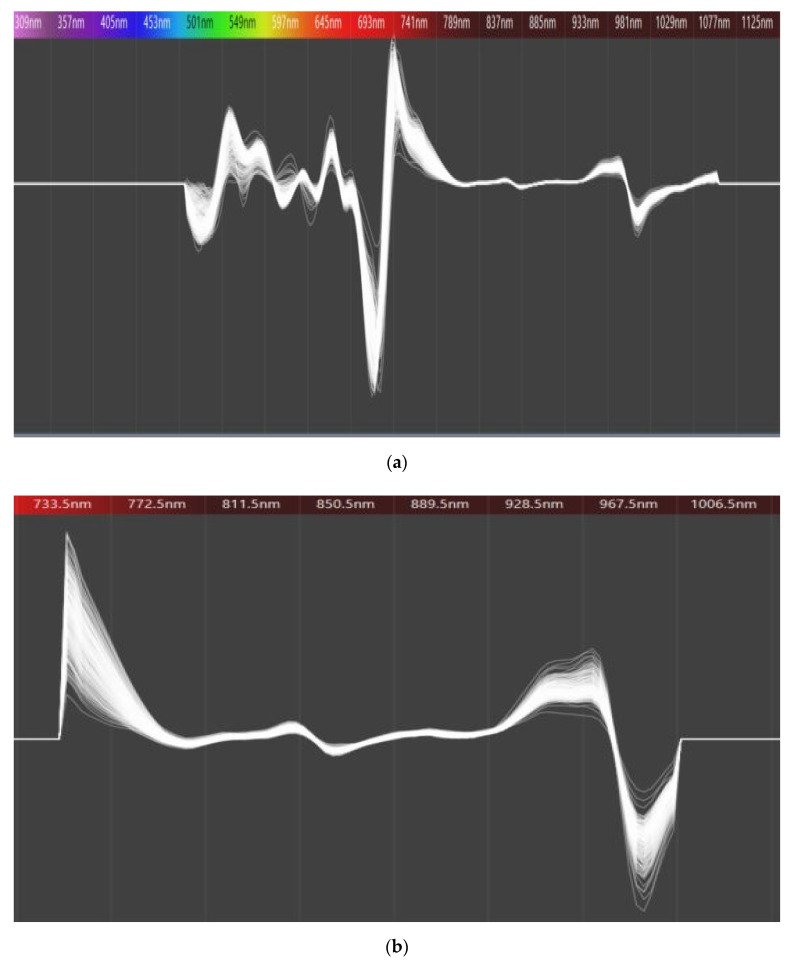
(**a**) Second derivative spectra of 350 hardy kiwi fruits at 300–1100 nm; and (**b**) 729–975 nm.

Figure 2a shows the second derivative spectra of hardy kiwi samples. The region covers Vis/NIR spectra range from 300 nm to1100 nm. According to Figure 2a, the optimal spectral range for model calibration of kiwi fruits was 729–975 nm, and this finding was in accordance with previous studies carried out to determine internal quality attributes of other fruits [48,49]. On the other hand, spectral profiles of all hardy kiwi samples exhibited a common pattern over the whole NIR region which indicates a similar component in all samples for area and species. The most important attributes affect the NIRS are chemical bonds, such as C-H, N-H, O-H, and C-O, which are subjected to stretching or bending caused by the vibrational energy change during irradiation by NIR light [50]. Outside the selected window of wavelength regions were noisy and uninformative due to the absorbance of chlorophyll and other pigments in the visible range of 400–700 nm [49]. From Figure 2b, the absorbance spectra were converted to the second derivative spectra and narrowed the wavelength region (729–975 nm) that could be used for the analysis of carbohydrate, sugar, and water absorbance bands in the NIR [51].

### 3.2. Statistical Properties

In this study, a total of 3128 samples were obtained from five areas of South Korea and were divided into two subsets equally as calibration and validation set with respect to each area, their species, and all together. Table 3, Table 4 and Table 5 show the summary statistics of actual SSC for all datasets selected for the calibration and validation of the models in each area, each species, and combined datasets, respectively. The SSC ranges of calibration and validation set between 5.0 and 27.2%, which indicated that calibration and validation sets covered a broad enough range.

### 3.3. Principal Component Analysis (PCA)

**Figure 3:**
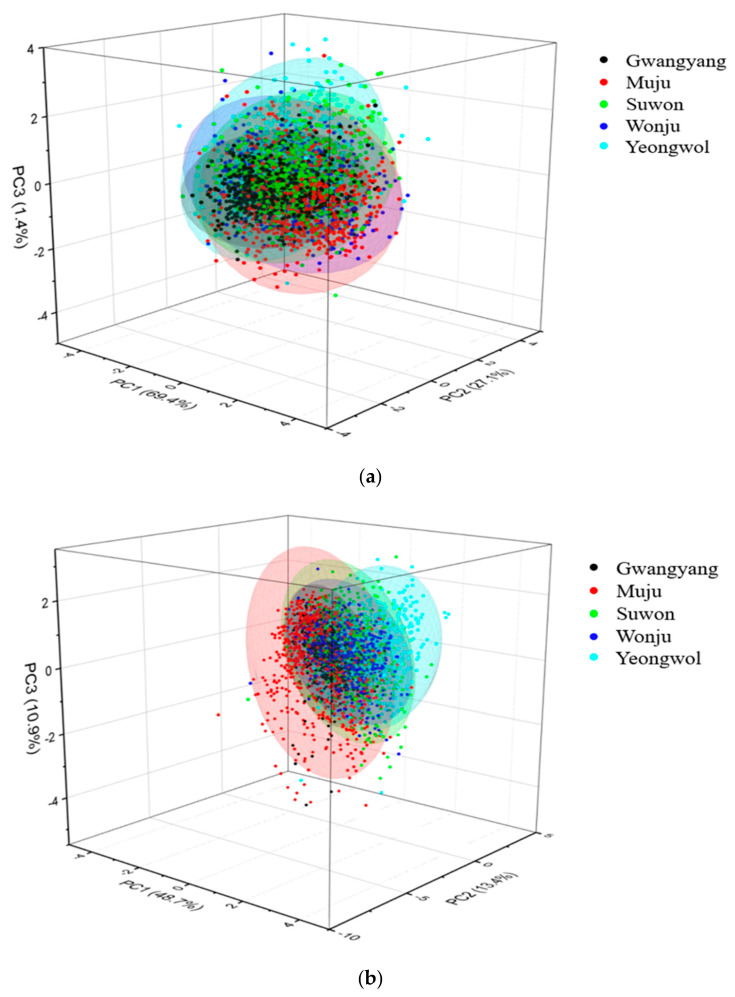
(**a**) Principal component analysis (PCA) score plot with original spectra data of Gwangyang, Muju, Suwon, Wonju, and Yeongwol areas (PC1 vs. PC2 vs. PC3); (**b**) PCA score plot with pre-treated spectra (MSC + SNV) of Gwangyang, Muju, Suwon, Wonju, and Yeongwol areas (PC1 vs. PC2 vs. PC3).

The PCA method was used to investigate the variability of dataset along with the spectral differences among the five-area datasets. Before PCA computation, second derivative data were pre-treated with the combination of MSC and SNV preprocessing because the combination was best suited among eight preprocessing techniques. Figure 3a,b illustrates the PCA score plots of PC1 vs. PC2 vs. PC3 of original and pre-treated spectra data. The results of PCA analysis with second derivative spectra attained PC1 values of 69.4%, PC2 of 27.1%, and PC3 by 1.4%. Where, PC1 is a component that explains the greatest variability in the data set, and PC2 and PC3 explain the remaining variability. After the pre-treatment, the values of PC1, PC2, and PC3 were found to be 48.7%, 13.4%, and 10.9%, respectively. Data from all the five areas are evenly scattered in the space that exhibited similar characteristics of fruits collected from different areas.

### 3.4. PCA Loadings

**Figure 4:**
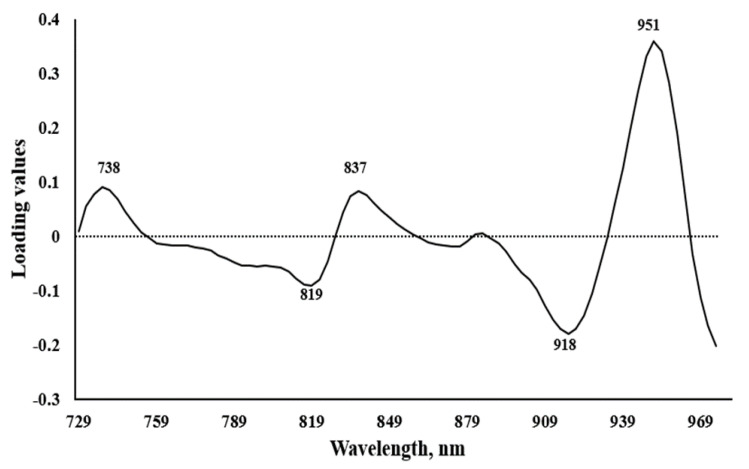
The first loading values of pretreated spectra.

To select the important and responsible wavelengths for the data grouping in PCA score plot, the pretreated spectra and the first loading were taken into consideration. From Figure 4, important wavelengths can be observed at 738, 819, 837, 843, 918, and 951 nm which correspond to the third overtones of C-H bond in water carbohydrates, starch, and sucrose, where peaks at 738, 837, and 951 nm are main and distinguishing [52]. This wavelength range is reliable for SSC prediction which is supported by instrument’s manual information for SSC and dry matter prediction of kiwi fruits.

### 3.5. PLS Models for the Prediction of SSC-Area Dataset

PLS models were developed using the pre-processed spectra in each area. Table 6 shows the best result of calibration and validation models among the eight preprocessing techniques from the spectral sampling range of 729–975 nm wavelengths. Other preprocessing results of the area using PLSR are provided as Supplementary Material (see Table S1).

**Figure 5:**
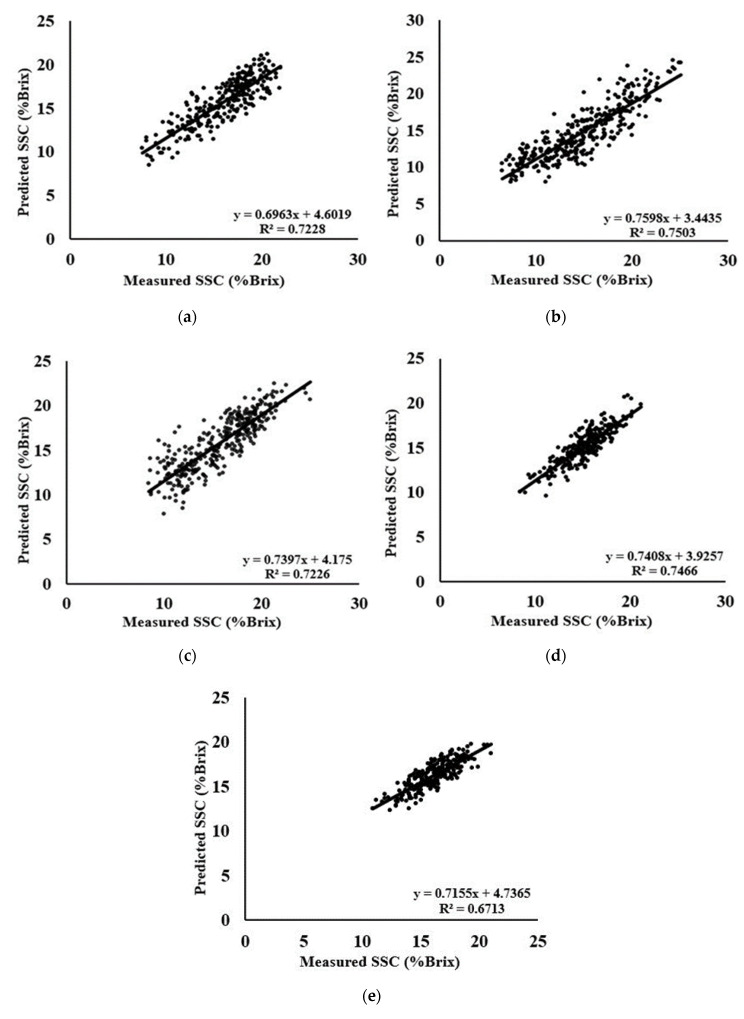
Scatter plots of measured versus predicted SSC using (**a**) second derivative; (**b**) MSC + SNV; (**c**) SNV; (**d**) MSC + OSC; and (**e**) MSC + SNV for G, M, S, W, and Y area, respectively.

The best predicted R^2^ of G, M, S, W, and Y areas were 0.72, 0.75, 0.72, 0.74, and 0.67, respectively obtained from different preprocessing with different latent variable (LV) numbers. Samples of Y area were found with relatively unsatisfactory results with all the preprocessing due to the negative impact of a number of samples on models. Although the same preprocessing was applied for all the areas, MSC + SNV was found excellent for M and Y area regarding the highest prediction correlation coefficient R^2^ of 0.75 and lowest RMSEP values of 2.0051. For the G area, no preprocessing was effective to increase the predictability except the original second derivative preprocessing with the highest predicted R^2^ of 0.72 and the lowest RMSEP of 1.7136. Regarding the S and W area, SNV and MSC + OSC suited most irrespective of others. However, preprocessing was applied to boost model performance by increasing the correlation coefficient and reducing RMSEP values, and in some cases, it was found that the latent variable (LV) numbers could be reduced by using data preprocessing. The SNV + OSC and MSC preprocessing significantly reduced the LV value that is irrespective of the calibration and prediction performance. It can be noted that different preprocessing techniques had effects on the prediction performance of the model using the PLS regression method. The best results within the number of preprocessing are shown in Figure 5 with the scattered plot of measured and predicted SSC for each area.

### 3.6. PLS Models for the Prediction of SSC-Species and Combine Dataset

Table 7 includes the best SSC model calibration and prediction results for each species data analyzed by using PLSR with the same preprocessing techniques and spectral sampling range. Species results with the other preprocessing techniques using PLSR are shown as Supplementary Material (see Table S2). Table 8 shows the combined data results using the same procedures.

**Figure 6:**
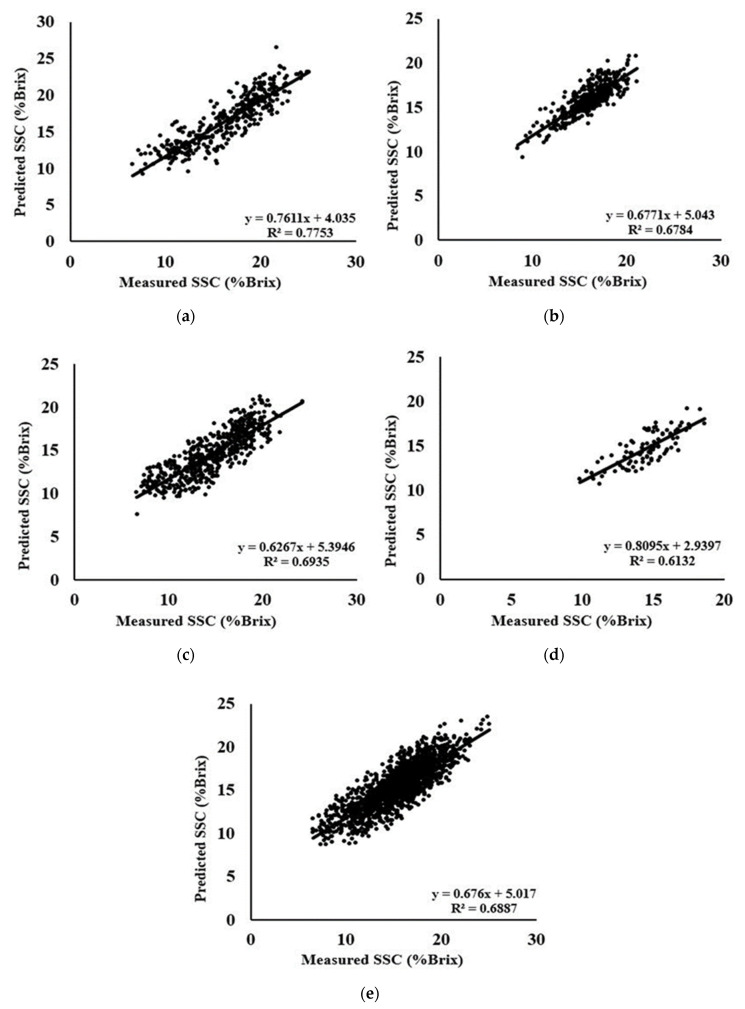
Scatter plots of measured versus predicted SSC using (**a**) Autoscale; (**b**) MSC + SNV; (**c**) Autoscale; (**d**) second derivative; and (**e**) MSC + OSC for A, C, D, Gb species and combine data, respectively.

Table 7 shows the predicted values of R^2^ of A, C, D, and Gb species were 0.77, 0.67, 0.69, and 0.61 with the best preprocessing of Autoscale, MSC + SNV, Autoscale, and second derivative, respectively. Species results of PLSR are unsatisfactory except species A because species A includes large data numbers and a mixture of different area farms. For species A, the value of LV was found 11 for Autoscale preprocessing and resulted in high RPD of 2.1411 with the lowest RMSEP of 1.8775. In the case of species D, PLSR was unable to predict calibration and prediction models with MSC + OSC preprocessing. The species Gb resulted in high calibration correlation coefficient but unable to perform satisfactorily in prediction condition because of having a few data. The combined dataset includes all data together, and it was expected to show lower performance compared to the area and species due to the adverse influence of some data from each area or species. In this case, MSC + OSC was found to be best among all the preprocessing with LV of 7, calibration correlation coefficient of 0.66, the prediction correlation coefficient of 0.68, RMSEP of 1.8286, and higher RPD of 1.8210. The scattered plot of best measured and predicted SSC is shown in Figure 6.

### 3.7. SVM-R Models for the Prediction of SSC-Area Dataset

Unlike PLSR, SVM-R also applied to the same data to compare the SSC predictability of two analyzing methods. The best predicted results of SVM-R analysis with the same preprocessing are presented in Table 9. Results of the area with other preprocessing techniques using SVM-R can be found in the Supplementary Material (see Table S3). The predicted R^2^ for G, M, S, W, and Y areas were 0.68, 0.80, 0.79, 0.75, and 0.69, respectively with the lowest RMSEP and highest RPD from Autoscale preprocessing.

**Figure 7:**
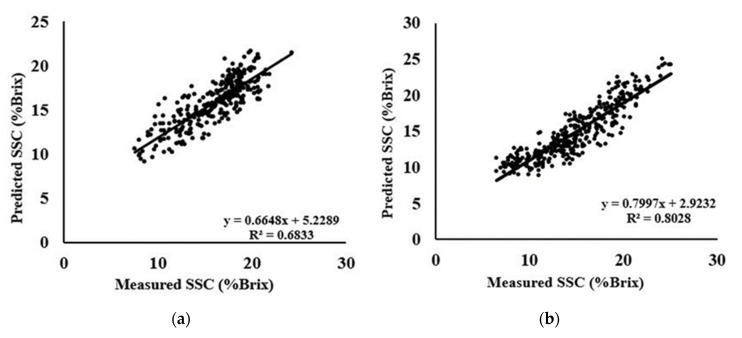

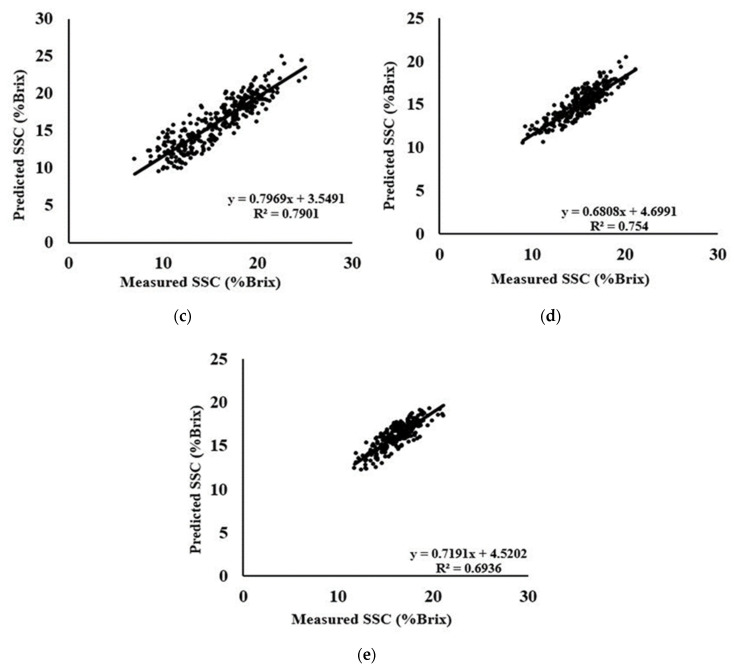
Scatter plots of measured versus predicted SSC using SVM-R method with Autoscale preprocessing (**a**) G; (**b**) M; (**c**) S; (**d**) S; and (**e**) Y area, respectively.

The SVM fits well for non-linear models and results in better calibration and prediction of soluble solids with higher R^2^, lowest RMSEP, and a higher ratio of prediction to deviation (RPD). Area M follows the same trend of good prediction ability as PLSR with a higher calibration and prediction correlation coefficient of 0.82 and 0.80, respectively. While the lowest RMSEP was 1.8421 and the highest RPD was 2.2691 for area M. Again, area Y showed a lower prediction correlation of 0.69 but resulted in a higher RPD value of 2.3883 where lower prediction ability of this area indicates an issue with the hardy kiwis regarding the adverse influence of samples on models. Data of area S and W were found to be satisfactory in every case with Autoscale preprocessing. Remarkably, second derivative data and MSC preprocessing on second derivative data showed very poor results for each area and the same models obtained with the SVM-R analysis yielded better calibration and prediction outcomes only with the Autoscale preprocessing except area G. The scattered plot of measured and predicted SSC is shown in Figure 7.

### 3.8. SVM Models for the Prediction of SSC- Species and Combine Dataset

Table 10 presents the best predicted results of SVM-R analysis obtained from the species, and results with the other preprocessing techniques are presented in the Supplementary Material (see Table S4). Table 11 presents the combine dataset results with all the preprocessing techniques. For the species table, the best-predicted correlation coefficient was obtained from Autoscale preprocessing for species A, C, and D with the value of 0.79, 0.73, and 0.71, respectively. Another species Gb showed poor prediction correlation of 0.62. In addition, Gb relies on MSC + SNV preprocessing with higher predictability, the lowest RMSEP and higher RPD among preprocessing techniques. The second derivative and the preprocessing MSC on second derivative data yielded poor results and followed the same trend for each area.

**Figure 8:**
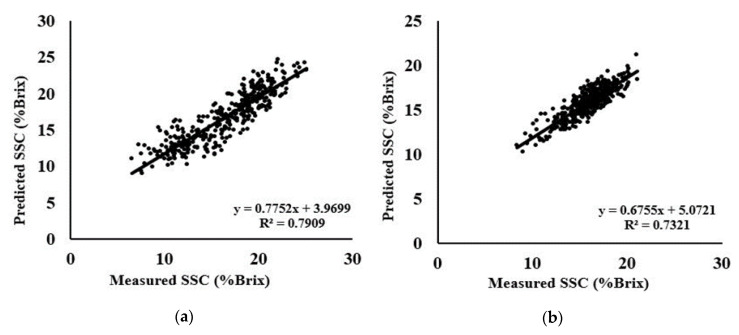

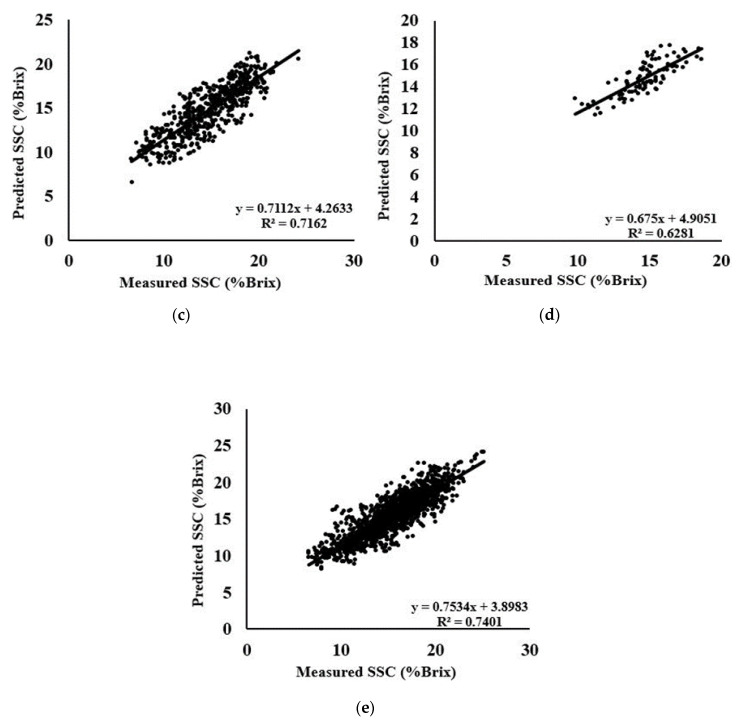
Scatter plots of measured versus predicted SSC using SVM-R method with Autoscale preprocessing for (**a**) A; (**b**) C; (**c**) D species; (**d**) MSC + SNV for Gb species; and (**e**) Autoscale for combine data.

On the other hand, for the combined dataset, Autoscale preprocessing had satisfactory results with a high correlation coefficient of 0.74, the lowest RMSEP of 1.6867, and a higher RPD value of 1.9742. The scattered plot of measured and predicted SSC is shown in Figure 8.

## 4. Discussion

### Comparison of PLSR and SVM-R Analysis

In this study, PLSR and SVMR analysis were performed on second derivative spectra data obtained from original/absorbance spectra data. Various pre-treatments (MSC, SNV, OSC, and Autoscale) and their combinations (SNV + OSC, MSC + SNV, and MSC + OSC) were applied to the second derivative data to obtain the best predictive model for the SSC quality analysis of hardy kiwi. Regarding the comparison between the PLSR and SVM-R, the models were developed on the various dataset obtained from the area, species, and combine.

From the PLSR results for the area, the best predicted correlation coefficient can be found between 0.67 and 0.75, where SVM-R performed better than the PLSR models with a correlation coefficient of 0.68 and 0.80. In both cases, area M showed the highest predicted correlation coefficient with the lowest RMSEP and higher RPD value that indicates the optimum models RPD ratio between 1.5 and 2.5 is suitable for screening purposes [53]. Furthermore, with SVM-R analysis, RPD values of optimum models were higher than the PLSR optimum models. That indicates more reliability of SVM-R regression method over PLSR. Notably, Autoscale preprocessing technique exhibited satisfactory results than any other preprocessing for SVM-R analysis. On the other hand, optimum results of PLSR analysis confides on various preprocessing techniques. The values of regression coefficient of SSC were slightly higher than those of 0.72 to 0.89 obtained by Escribano et al. [48] for the prediction of SSC of sweet cherries. Furthermore, a study, conducted on peppers using a portable Vis/NIR spectrometer showed the prediction correlation coefficient of 0.68, SEP of 1.24, and RPD of 1.78 for SSC prediction [53].

On the other hand, species A was found superior to other species with Autoscale preprocessing for both methods resulting in the best prediction correlation coefficient of 0.77 and 0.79, and higher RPD of 2.1411 and 2.1896. This result also indicates the higher reliability of the SVM-R method for species A. For species C, SVM-R follows the same pattern for the prediction of SSC. Here, Autoscale preprocessing demonstrated a higher prediction possibility with a higher correlation coefficient of 0.73 and higher RPD. On the other hand, PLS resulted in the highest prediction R^2^ and RPD of 0.67 and 1.7917, respectively with MSC + SNV preprocessing. Like species A, species D followed the same pattern and showed satisfactory prediction capability with the lowest RMSEP and higher RPD for Autoscale preprocessing by the SVM-R method. In contrast, the prediction correlation coefficient of PLSR was below 0.70, whereas MSC + OSC preprocessing was unable to perform the prediction. Feng et al. studied on *Actinidia chinesis* “Hort16A” for the prediction of SSC and observed the prediction of R^2^ with 0.72 and RMSEP of 1.28 [54]. Again, Kim et al. [33] reported the prediction R^2^ of 0.72 for the SSC prediction of baby kiwi fruit.

The last species Gb showed unsatisfactory results with both PLSR and SVM-R analysis (R^2^ of 0.61 and 0.62, respectively) among the preprocessing techniques. This might be due to the inadequate number of samples involved in the calibration and prediction and adverse influence of samples on model. External factors, such as temperature, peel, humidity, product morphology, etc., can affect the model performance because of the high errors in data collection [53]. For the prediction of the combined dataset, SVM-R performed quite a satisfactory result compared to PLSR. The PLSR analysis resulted in a prediction correlation coefficient of 0.68 with the lowest RMSEP of 1.8286 and a higher RPD of 1.8210 with MSC + OSC preprocessing. Notably, Autoscale preprocessing of SVM-R analysis increased the prediction capability with a value of 0.74, minimum RMSEP of 1.6867 and higher RPD of 1.9742. In a study using d’Anjou and Bartlett pear, the predicted R^2^ of the validation dataset for SSC prediction was in the ranges of 0.651–0.844 [49]. In a further study, the standard error of laboratory (SEL) of reference method will be calculated to determine the SEP/SEL ratio that evaluates the predictive ability of equations and the precision level of models for accurate routine use.

The second derivative preprocessing technique showed the best performance in prediction for area G and species Gb among prediction results of area and species. Contrarily, SVM-R analysis datasets with no preprocessing on second derivative data do not follow the same pattern as PLSR. Furthermore, Autoscale preprocessing is acceptable in most of the cases for SVM-R analysis.

## 5. Conclusions

The study was conducted to evaluate the application of a handheld portable Vis/NIR spectrometer for the SSC prediction using different preprocessing techniques with two analyzing methods. Hardy kiwi fruits were collected from five different areas of South Korea that include four different species. It has been shown that handheld Vis/NIRS is a promising tool for predicting the internal quality attributes of hardy kiwi fruits. Furthermore, it was found that Vis/NIRS with the combination of chemometrics could drive to proper models by choosing an appropriate preprocessing technique and analyzing method.

PLSR and SVM-R models were compared to each analysis method. Different preprocessing techniques were best for the different areas, species, and combined dataset model development using PLSR, whereas Autoscale preprocessing was found best in maximum cases for the model development using SVM-R analysis. Moreover, SVM-R analysis with Autoscale preprocessing resulted in better prediction ability with the higher correlation coefficient, lower RMSEP, and higher RPD value except for Gwangyang (G) area. The PLSR method is one of the common and widely used techniques in chemometrics, but relatively low prediction ability was obtained for each sector of datasets because of the use of a large number of samples containing very few adverse and influential samples. Results revealed that SVM-R analysis with Autoscale preprocessing can be used to validate the model and to predict the SSC of hardy kiwi fruit. The SSC prediction of hardy kiwi fruits using NIR data revealed that the use of a handheld portable spectrometer, such as F-750 produce quality meter shows excellency for the analysis of internal quality parameters of fruits. However, the RBF-SVM uses the assumption that there is an analytical function that describes the hyperplane. In the future, another regression model such as random forest model will be presented that does not use any assumption regarding hyperplane. Furthermore, models can be developed using Model builder software to check the instrument off the shelf capability to predict any other internal quality attributes of hardy kiwi fruits.

## Figures and Tables

**Table 1:** Location of each farm of five areas.

Area	Farms	Latitude, Longitude
Gwangyang	G	35° 1.972′ N, 127° 35.505′ E
Muju	M1	36° 0.230′ N, 127° 40.295′ E
	M2	35° 55.788′ N, 127° 42.389′ E
	M3	36° 0.374′ N, 127° 44.935′ E
Suwon	S1	37° 15.038′ N, 126° 57.483′ E
	S2	37° 15.876′ N, 126° 55.477′ E
Wonju	W1	37° 27.046′ N, 127° 53.167′ E
	W2	37° 22.42′ N, 128° 0.502′ E
	W3	35° 55.784′ N, 127° 42.699′ E
Yeongwol	Y1	37° 12.23′ N, 128° 36.622′ E

**Table 2:** Compositions of area, species, and combined datasets.

Name	Data Set	Data Set Composition
Area	Gwangyang (G)	G
	Muju (M)	M_1_, M_2_, M_3_
	Suwon (S)	S_1_, S_2_
	Wonju (W)	W_1_, W_2_, W_3_
	Yeongwol (Y)	Y
Species	Autumn sense (A)	M_1_, S_1_, S_2_
	Chungsan (C)	W_2_, W_3_, Y
	Daesung (D)	G, M_2_, M_3_
	Green ball (Gb)	W_3_
Combined data		G, M_1_, M_2_, M_3_, S_1_, S_2_ W_1_, W_2_, W_3_, Y

**Table 3:** Statistical properties of soluble solids content (SSC) in each area.

Characteristic	Area	Sample Set	Mean	SD	Median	Max.	Min.	No. of Samples
SSC	Gwangyang (G)	Calibration	16.17	3.33	16.8	23.7	7.7	300
		Validation	16.04	3.26	16.85	24.2	7.5	300
	Muju (M)	Calibration	14.57	4.10	14.3	27.2	6.6	353
		Validation	14.82	4.18	14.75	25.1	5.0	352
	Suwon (S)	Calibration	15.67	3.83	16.1	23.0	6.5	343
		Validation	15.81	3.60	16.4	25.0	7.0	343
	Wonju (W)	Calibration	15.10	2.27	15.1	21.9	8.5	280
		Validation	15.3	2.32	15.5	21.1	8.4	279
	Yeongwol (Y)	Calibration	16.08	1.83	16.2	20.3	10.1	289
		Validation	16.14	2.00	16.2	21.3	7.3	289

**Table 4:** Statistical properties of SSC for each species.

Characteristic	Species	Sample Set	Mean	SD	Median	Max.	Min.	No. of Samples
SSC	Autumn sense (A)	Calibration	16.50	3.84	17.0	27.2	7.0	405
		Validation	16.39	4.02	17.0	25.1	6.5	404
	Chungsan (C)	Calibration	15.86	2.21	16.1	21.9	7.3	467
		Validation	15.92	2.12	16.0	21.1	8.4	466
	Daesung (D)	Calibration	14.93	3.63	15.2	23.7	5	591
		Validation	14.71	3.50	15.0	24.2	6.6	591
	Green ball (Gb)	Calibration	14.67	1.83	14.95	19.0	10.0	102
		Validation	14.56	1.82	14.7	18.6	9.8	102

**Table 5:** Statistical properties of SSC for combine data.

Characteristic	All Together	Sample Set	Mean	SD	Median	Max.	Min.	No. of Samples
SSC	Combine	Calibration	15.56	3.27	15.8	27.2	6.6	1564
		Validation	15.54	3.33	15.8	25.1	5.0	1564

**Table 6:** Best results of each area with second derivative data and their preprocessing at a spectral sampling range of 729–975 nm using partial least square regression (PLSR).

Area	Pre-Processing	LV	Calibration		Prediction (Validation)			RPD
			R^2^	RMSEC	R^2^	RMSEP	Bias	
G	Second derivative	6	0.71	1.7832	0.72	1.7136	−0.28473	1.9024
M	MSC + SNV	8	0.759	2.016	0.750	2.0846	−0.12002	2.0051
S	SNV	9	0.718	2.0371	0.723	1.8521	0.068707	1.9437
W	MSC + OSC	4	0.835	0.92495	0.747	1.1514	−0.041329	2.0149
Y	MSC + SNV	11	0.662	1.0695	0.671	1.0343	0.16688	1.9336

**Table 7:** Best results of species with second derivative data and their preprocessing at a spectral sampling range of 729–975 nm using PLSR.

Species	Pre-Processing	LV	Calibration		Prediction (Validation)			RPD
			R^2^	RMSEC	R^2^	RMSEP	Bias	
A	Autoscale	11	0.775	1.8256	0.775	1.8775	0.090077	2.1411
C	MSC + SNV	10	0.662	1.2857	0.678	1.1832	−0.11288	1.7917
D	Autoscale	7	0.659	2.1201	0.694	1.9648	−0.085569	1.7813
Gb	Second derivative	8	0.769	0.88278	0.613	1.2167	0.16661	1.4958

**Table 8:** PLSR results of combine data with second derivative and their preprocessing at a spectral sampling range of 729–975 nm.

All Together	Pre-Processing	LV	Calibration		Prediction (Validation)			RPD
			R^2^	RMSEC	R^2^	RMSEP	Bias	
Combine	Second derivative	11	0.652	1.9338	0.651	1.9512	0.018924	1.7066
	MSC	10	0.656	1.9233	0.667	1.9049	−0.027812	1.7481
	SNV	10	0.657	1.9201	0.665	1.9107	−0.021354	1.7428
	OSC	4	0.654	1.9305	0.651	1.9364	0.02903	1.7196
	SNV + OSC	7	0.663	1.9033	0.674	1.8786	−0.015084	1.7725
	MSC + SNV	7	0.632	1.9884	0.633	2.0067	−0.020487	1.6594
	MSC + OSC	7	0.662	1.9056	0.689	1.8286	−0.019629	1.8210
	Autoscale	10	0.652	1.934	0.669	1.887	0.025924	1.7647

**Table 9:** Best results of each area with second derivative data and their preprocessing at a spectral sampling range of 729–975 nm using support vector machine regression (SVM-R).

Area	Pre-Processing	Calibration		Prediction (Validation)			RPD
		R^2^	RMSEC	R^2^	RMSEP	Bias	
G	Autoscale	0.706	1.8103	0.683	1.8424	−0.146	1.7694
M	Autoscale	0.823	1.7344	0.803	1.8421	−0.064418	2.2691
S	Autoscale	0.802	1.7238	0.790	1.6644	0.32509	2.1629
W	Autoscale	0.792	1.0415	0.754	1.1596	−0.18288	2.0006
Y	Autoscale	0.755	0.91677	0.694	0.9714	−0.008294	2.3883

**Table 10:** Best results of species with second derivative data and their preprocessing at a spectral sampling range of 729–975 nm using SVM-R.

Species	Pre-Processing	Calibration		Prediction (Validation)			RPD
		R^2^	RMSEC	R^2^	RMSEP	Bias	
A	Autoscale	0.776	1.834	0.791	1.8359	0.26706	2.1896
C	Autoscale	0.706	1.2039	0.732	1.102	−0.09689	1.9237
D	Autoscale	0.783	1.6942	0.716	1.8368	−0.009629	1.9054
Gb	MSC + SNV	0.652	1.0846	0.628	1.1143	0.15616	1.6333

**Table 11:** SVM-R results of combine data with second derivative and their preprocessing at a spectral sampling range of 729–975 nm.

All Together	Pre-Processing	Calibration		Prediction (Validation)			RPD
		R^2^	RMSEC	R^2^	RMSEP	Bias	
Combine	Second derivative	0.101	3.183	0.095	3.2118	0.19047	1.0368
	MSC	0.183	3.2424	0.188	3.268	0.25516	1.0189
	SNV	0.728	1.7104	0.657	1.9596	0.063723	1.6993
	OSC	0.533	3.157	0.490	3.2216	0.24963	1.0336
	SNV + OSC	0.809	1.4343	0.664	1.9452	−0.0092358	1.7119
	MSC + SNV	0.728	1.7104	0.657	1.9595	0.063723	1.6994
	MSC + OSC	0.397	3.2445	0.371	3.3063	0.27998	1.0071
	Autoscale	0.766	1.5864	0.740	1.6867	0.063042	1.9742

## Supplementary Materials

The following are available online at https://www.mdpi.com/2304-8158/9/8/1078/s1, Table S1: PLSR results (excluding best) of each area with second derivative data and their preprocessing techniques applied at a spectral sampling range of 729–975 nm, Table S2: PLSR results (excluding best) of each species with second derivative data and their preprocessing techniques applied at a spectral sampling range of 729–975 nm, Table S3: SVM-R results (excluding best) of each area with second derivative data and their preprocessing techniques applied at a spectral sampling range of 729–975 nm, Table S4: SVM-R results (excluding best) of each species with second derivative data and their preprocessing techniques applied at a spectral sampling range of 729–975 nm.
