# Supplementary material for: A Comparative Study of PLSR and SVM-R with Various Preprocessing Techniques for the Quantitative Determination of Soluble Solids Content of Hardy Kiwi Fruit by a Portable Vis/NIR Spectrometer
Source: Foods. 2020 Aug 7;9(8):1078. doi: 10.3390/foods9081078 (PMC7466312; doi:10.3390/foods9081078)
Supplement: Supplementary file 1 [file foods-09-01078-s001.pdf]

**Supplementary material**

**Table S1.** PLSR results (excluding best) of each area with second derivative data and their preprocessing techniques applied at a spectral sampling range of 729-975 nm.

| Area | Pre-processing | LV | Calibration | | Prediction (Validation) | | | RPD |
|---|---|---|---|---|---|---|---|---|
| | | | $R^2$ | RMSEC | $R^2$ | RMSEP | Bias | |
| G | MSC | 9 | 0.69 | 1.8564 | 0.606 | 2.0205 | -0.13652 | 1.6134 |
| | SNV | 9 | 0.69 | 1.8429 | 0.600 | 2.0462 | -0.14782 | 1.5931 |
| | OSC | 5 | 0.74 | 1.7009 | 0.716 | 1.7221 | -0.25055 | 1.8930 |
| | SNV+OSC | 3 | 0.730 | 1.7322 | 0.604 | 1.9634 | -0.22866 | 1.6603 |
| | MSC+SNV | 9 | 0.695 | 1.8429 | 0.614 | 1.9972 | -0.082259 | 1.6322 |
| | MSC+OSC | 3 | 0.729 | 1.736 | 0.598 | 1.9789 | -0.20336 | 1.6473 |
| | Autoscale | 11 | 0.733 | 1.724 | 0.631 | 1.9122 | -0.25639 | 1.7048 |
| M | Second derivative | 8 | 0.758 | 2.0197 | 0.729 | 2.1683 | -0.06423 | 1.9277 |
| | MSC | 8 | 0.75 | 2.02 | 0.71 | 2.24 | -0.021835 | 1.8660 |
| | SNV | 8 | 0.759 | 2.016 | 0.716 | 2.236 | -0.022298 | 1.8694 |
| | OSC | 4 | 0.767 | 1.9801 | 0.686 | 2.3656 | 0.056206 | 1.7669 |
| | SNV+OSC | 4 | 0.745 | 2.0732 | 0.712 | 2.2541 | -0.090836 | 1.8543 |
| | MSC+OSC | 5 | 0.761 | 2.0073 | 0.745 | 2.1165 | -0.19011 | 1.9749 |
| | Autoscale | 6 | 0.754 | 2.0347 | 0.745 | 2.098 | -0.020732 | 1.9923 |
| S | Second derivative | 9 | 0.732 | 1.9828 | 0.731 | 1.8248 | 0.05416 | 1.9728 |
| | MSC | 9 | 0.717 | 2.0395 | 0.722 | 1.8561 | 0.068057 | 1.9395 |
| | OSC | 3 | 0.769 | 1.8442 | 0.676 | 2.0299 | 0.064776 | 1.7734 |
| | SNV+OSC | 6 | 0.757 | 1.8883 | 0.704 | 1.9004 | 0.070428 | 1.8943 |
| | MSC+SNV | 5 | 0.718 | 2.0371 | 0.717 | 1.8801 | 0.035105 | 1.9147 |
| | MSC+OSC | 6 | 0.757 | 1.8908 | 0.693 | 1.9232 | 0.021 | 1.8718 |
| | Autoscale | 6 | 0.705 | 2.0802 | 0.685 | 1.9663 | -0.1403 | 1.8308 |
| W | Second derivative | 10 | 0.792 | 1.0382 | 0.716 | 1.2195 | -0.13191 | 1.9024 |
| | MSC | 9 | 0.798 | 1.0244 | 0.739 | 1.1785 | -0.071407 | 1.9686 |
| | SNV | 9 | 0.798 | 1.0231 | 0.739 | 1.1775 | -0.06994 | 1.9702 |
| | OSC | 3 | 0.818 | 0.97231 | 0.718 | 1.1578 | -0.065662 | 2.0038 |
| | SNV+OSC | 4 | 0.835 | 0.92454 | 0.745 | 1.1609 | -0.0459 | 1.9984 |
| | MSC+SNV | 9 | 0.798 | 1.0231 | 0.743 | 1.1772 | -0.065775 | 1.9707 |
| | Autoscale | 6 | 0.775 | 1.0797 | 0.740 | 1.1741 | -0.069919 | 1.9759 |
| Y | Second derivative | 6 | 0.597 | 1.1676 | 0.668 | 1.0445 | 0.12916 | 1.9147 |
| | MSC | 12 | 0.666 | 1.0622 | 0.642 | 1.1003 | 0.11768 | 1.8176 |

| | | | | | | | |
|---|---|---|---|---|---|---|---|
| SNV | 12 | 0.667 | 1.0615 | 0.653 | 1.0544 | 0.1624 | 1.8968 |
| OSC | 5 | 0.631 | 1.1169 | 0.643 | 1.0594 | 0.14955 | 1.8878 |
| SNV+OSC | 3 | 0.577 | 1.1956 | 0.590 | 1.1634 | 0.066877 | 1.7190 |
| MSC+OSC | 3 | 0.573 | 1.2016 | 0.539 | 1.2336 | 0.0090186 | 1.6212 |
| Autoscale | 7 | 0.637 | 1.1082 | 0.634 | 1.1202 | 0.13131 | 1.7853 |

**Table S2.** PLSR results (excluding best) of each species with second derivative data and their preprocessing techniques applied at a spectral sampling range of 729-975 nm.

| Area | Pre-processing | LV | Calibration | | Prediction (Validation) | | | RPD |
|---|---|---|---|---|---|---|---|---|
| | | | $R^2$ | RMSEC | $R^2$ | RMSEP | Bias | |
| | Second derivative | 10 | 0.769 | 1.8494 | 0.729 | 2.0988 | 0.1389 | 1.9153 |
| | MSC | 9 | 0.764 | 1.8679 | 0.716 | 2.1484 | 0.12393 | 1.8711 |
| | SNV | 10 | 0.769 | 1.8485 | 0.713 | 2.1504 | 0.1583 | 1.8694 |
| A | OSC | 5 | 0.789 | 1.7665 | 0.692 | 2.2481 | 0.2215 | 1.7881 |
| | SNV+OSC | 8 | 0.788 | 1.7727 | 0.692 | 2.2444 | 0.1822 | 1.7911 |
| | MSC+SNV | 8 | 0.756 | 1.8989 | 0.761 | 1.9305 | 0.02984 | 2.0823 |
| | MSC+OSC | 5 | 0.780 | 1.8058 | 0.761 | 1.907 | -0.011216 | 2.1080 |
| | Second derivative | 7 | 0.650 | 1.3097 | 0.653 | 1.217 | -0.11502 | 1.7419 |
| | MSC | 10 | 0.661 | 1.2874 | 0.678 | 1.1836 | -0.10319 | 1.7911 |
| | SNV | 10 | 0.662 | 1.2857 | 0.678 | 1.1832 | -0.11288 | 1.7917 |
| C | OSC | 2 | 0.648 | 1.3122 | 0.602 | 1.2589 | -0.1233 | 1.6840 |
| | SNV+OSC | 2 | 0.581 | 1.4319 | 0.577 | 1.2415 | -0.16554 | 1.7076 |
| | MSC+OSC | 2 | 0.584 | 1.4276 | 0.591 | 1.3606 | -0.058465 | 1.5581 |
| | Autoscale | 7 | 0.650 | 1.3097 | 0.653 | 1.217 | -0.11502 | 1.7419 |
| | Second derivative | 6 | 0.655 | 2.1316 | 0.645 | 2.0995 | -0.06941 | 1.6670 |
| | MSC | 9 | 0.668 | 2.0904 | 0.615 | 2.1847 | -0.03754 | 1.6020 |
| D | SNV | 9 | 0.670 | 2.0862 | 0.621 | 2.1696 | -0.03649 | 1.6132 |
| | OSC | 3 | 0.689 | 2.0239 | 0.648 | 2.0937 | -0.086748 | 1.6716 |
| | SNV+OSC | 5 | 0.689 | 2.025 | 0.675 | 2.0252 | -0.070331 | 1.7282 |
| | MSC+SNV | 9 | 0.670 | 2.0862 | 0.621 | 2.1696 | -0.03649 | 1.6132 |
| | MSC+OSC | -- | -- | -- | -- | -- | -- | |
| | MSC | 8 | 0.766 | 0.8894 | 0.590 | 1.2764 | 0.13449 | 1.4258 |
| Gb | SNV | 8 | 0.765 | 0.89074 | 0.588 | 1.2834 | 0.1466 | 1.4181 |
| | OSC | 3 | 0.578 | 1.1929 | 0.492 | 1.3111 | 0.129 | 1.3881 |
| | SNV+OSC | 2 | 0.773 | 0.8749 | 0.486 | 1.3304 | -0.10542 | 1.3680 |

| | | | | | | | | |
|---|---|---|---|---|---|---|---|---|
| | MSC+SNV | 8 | 0.765 | 0.8904 | 0.588 | 1.3721 | 0.1466 | 1.3264 |
| | MSC+OSC | 2 | 0.781 | 0.85955 | 0.511 | 1.3167 | -0.13562 | 1.3822 |
| | Autoscale | 5 | 0.713 | 0.9848 | 0.581 | 1.2257 | 0.11367 | 1.4848 |

**Table S3.** SVM-R results (excluding best) of each area with second derivative data and their preprocessing techniques applied at a spectral sampling range of 729-975 nm.

| Area | Pre-processing | Calibration | | Prediction (Validation) | | | RPD |
|---|---|---|---|---|---|---|---|
| | | $R^2$ | RMSEC | $R^2$ | RMSEP | Bias | |
| G | Second derivative | 0.109 | 3.3544 | 0.080 | 3.173 | -0.28581 | 1.0274 |
| | MSC | 0.043 | 3.3619 | 0.135 | 3.1991 | -0.41735 | 1.0190 |
| | SNV | 0.680 | 1.8877 | 0.647 | 1.92 | -0.089892 | 1.6979 |
| | OSC | 0.680 | 1.8877 | 0.652 | 1.8704 | -0.012024 | 1.7429 |
| | SNV+OSC | 0.744 | 1.6929 | 0.576 | 2.1178 | -0.17743 | 1.5393 |
| | MSC+SNV | 0.680 | 1.8877 | 0.662 | 1.8505 | -0.036248 | 1.7616 |
| | MSC+OSC | 0.631 | 3.362 | 0.584 | 3.1789 | -0.16892 | 1.0255 |
| M | Second derivative | 0.125 | 4.0841 | 0.187 | 4.184 | -0.5525 | 0.9990 |
| | MSC | 0.153 | 4.092 | 0.196 | 4.192 | -0.50809 | 0.9971 |
| | SNV | 0.790 | 1.8862 | 0.735 | 2.1549 | 0.0054082 | 1.9397 |
| | OSC | 0.637 | 4.0168 | 0.625 | 4.1202 | -0.5015 | 1.0145 |
| | SNV+OSC | 0.913 | 1.2185 | 0.742 | 2.1428 | 0.031253 | 1.9507 |
| | MSC+SNV | 0.790 | 1.8862 | 0.735 | 2.1549 | 0.0054082 | 1.9397 |
| | MSC+OSC | 0.606 | 4.0938 | 0.625 | 4.1963 | -0.51838 | 0.9961 |
| S | Second derivative | 0.087 | 3.8507 | 0.096 | 3.6156 | 0.23571 | 0.9956 |
| | MSC | 0.084 | 3.8364 | 0.069 | 3.6227 | -0.3496 | 0.9937 |
| | SNV | 0.744 | 1.9402 | 0.702 | 1.9761 | 0.083213 | 1.8217 |
| | OSC | 0.715 | 3.8132 | 0.610 | 3.6007 | -0.34029 | 0.9998 |
| | SNV+OSC | 0.819 | 1.6444 | 0.660 | 2.1141 | 0.18773 | 1.7028 |
| | MSC+SNV | 0.744 | 1.9402 | 0.738 | 1.8381 | 0.070549 | 1.9585 |
| | MSC+OSC | 0.513 | 3.8331 | 0.575 | 3.5449 | -0.40904 | 1.0155 |
| W | Second derivative | 0.146 | 2.2486 | 0.111 | 2.3165 | -0.24406 | 1.0015 |
| | MSC | 0.102 | 2.2694 | 0.089 | 2.3378 | -0.2839 | 0.9923 |
| | SNV | 0.832 | 0.93505 | 0.731 | 1.2151 | -0.13596 | 1.9093 |
| | OSC | 0.815 | 2.2402 | 0.696 | 2.2982 | -0.12217 | 1.0094 |
| | SNV+OSC | 0.829 | 0.94466 | 0.731 | 1.2119 | -0.064282 | 1.9143 |
| | MSC+SNV | 0.832 | 0.93505 | 0.731 | 1.2151 | -0.13596 | 1.9093 |

| | | | | | | | |
|---|---|---|---|---|---|---|---|
| | MSC+OSC | 0.767 | 2.2643 | 0.719 | 2.3202 | -0.12973 | 0.9999 |
| Y | Second derivative | 0.189 | 1.8375 | 0.115 | 1.8966 | -0.11227 | 1.0545 |
| | MSC | 0.314 | 1.838 | 0.290 | 1.8305 | -0.15184 | 1.2674 |
| | SNV | 0.711 | 0.99131 | 0.574 | 1.2239 | 0.032897 | 1.8955 |
| | OSC | 0.197 | 1.8345 | 0.165 | 1.8687 | -0.14332 | 1.2415 |
| | SNV+OSC | 0.731 | 0.95736 | 0.555 | 1.2484 | 0.050385 | 1.8583 |
| | MSC+SNV | 0.711 | 0.99131 | 0.582 | 1.2251 | 0.023887 | 1.8937 |
| | MSC+OSC | 0.518 | 1.836 | 0.541 | 1.8286 | -0.15368 | 1.2687 |

**Table S4.** SVM-R results (excluding best) of each species with second derivative data and their preprocessing techniques applied at a spectral sampling range of 729-975 nm.

| Species | Pre-processing | Calibration | | Prediction (Validation) | | | RPD |
|---|---|---|---|---|---|---|---|
| | | $R^2$ | RMSEC | $R^2$ | RMSEP | Bias | |
| A | Second derivative | 0.152 | 3.7976 | 0.148 | 3.9642 | -0.26817 | 1.0140 |
| | MSC | 0.272 | 3.8548 | 0.255 | 4.0217 | -0.26231 | 0.9995 |
| | SNV | 0.793 | 1.757 | 0.732 | 2.104 | 0.29073 | 1.9106 |
| | OSC | 0.542 | 3.8049 | 0.487 | 3.9739 | -0.26156 | 1.0116 |
| | SNV+OSC | 0.813 | 1.6717 | 0.714 | 2.1696 | 0.28779 | 1.8528 |
| | MSC+SNV | 0.764 | 1.8783 | 0.761 | 1.9602 | 0.217 | 2.0508 |
| | MSC+OSC | 0.555 | 3.8643 | 0.494 | 4.031 | -0.25156 | 0.9972 |
| C | Second derivative | 0.112 | 2.1797 | 0.126 | 2.0892 | 0.08798 | 1.0147 |
| | MSC | 0.167 | 2.1987 | 0.166 | 2.1084 | 0.10606 | 1.0055 |
| | SNV | 0.680 | 1.2555 | 0.679 | 1.2145 | -0.11531 | 1.7455 |
| | OSC | 0.466 | 2.1878 | 0.466 | 2.0987 | 0.11121 | 1.0101 |
| | SNV+OSC | 0.753 | 1.1121 | 0.708 | 1.1326 | -0.10179 | 1.8717 |
| | MSC+SNV | 0.680 | 1.255 | 0.679 | 1.2145 | -0.11531 | 1.7455 |
| | MSC+OSC | 0.584 | 2.2004 | 0.592 | 2.1105 | 0.10309 | 1.0045 |
| D | Second derivative | 0.058 | 3.6056 | 0.015 | 3.4935 | 0.058253 | 1.0018 |
| | MSC | 0.040 | 3.6221 | 0.022 | 3.5005 | 0.092636 | 0.9998 |
| | SNV | 0.706 | 1.9712 | 0.650 | 2.077 | 0.088752 | 1.6851 |
| | OSC | 0.531 | 3.6116 | 0.442 | 3.4887 | 0.057618 | 1.0032 |
| | SNV+OSC | 0.724 | 1.9095 | 0.655 | 2.0502 | 0.024221 | 1.7071 |
| | MSC+SNV | 0.706 | 1.9712 | 0.652 | 2.0603 | 0.080017 | 1.6987 |
| | MSC+OSC | 0.555 | 3.6258 | 0.538 | 3.5075 | 0.067462 | 0.9978 |
| Gb | Second derivative | 0.046 | 1.8351 | 0.048 | 1.8293 | 0.16786 | 0.9949 |

| | | | | | | |
|---|---|---|---|---|---|---|
| MSC | 0.053 | 1.8362 | 0.053 | 1.8304 | 0.17298 | 0.9943 |
| SNV | 0.651 | 1.0846 | 0.458 | 1.3884 | 0.23218 | 1.3108 |
| OSC | 0.056 | 1.835 | 0.064 | 1.8287 | 0.16313 | 0.9952 |
| SNV+OSC | 0.906 | 0.58084 | 0.439 | 1.6089 | 0.22113 | 1.1312 |
| MSC+OSC | 0.743 | 1.8386 | 0.488 | 1.8579 | 0.13161 | 0.9796 |
| Autoscale | 0.707 | 1.0001 | 0.621 | 1.1516 | 0.18293 | 1.5804 |
